# Loneliness before and during the COVID-19 pandemic—are unpartnered and childless older adults at higher risk?

**DOI:** 10.1007/s10433-022-00718-x

**Published:** 2022-07-19

**Authors:** Bruno Arpino, Christine A. Mair, Nekehia T. Quashie, Radoslaw Antczak

**Affiliations:** 1 University of Florence, Viale Morgagni, 59, 50134 Florence, Italy; 2 University of Maryland, Baltimore County, USA; 3 University of Rhode Island, Kingston, USA; 4 SGH Warsaw School of Economics, Warsaw, Poland

**Keywords:** Loneliness, Partnership, Parenthood, Coronavirus, SHARE

## Abstract

COVID-19 mitigation efforts had the potential to exacerbate loneliness among older adults, particularly for the unpartnered or childless, yet specific studies on loneliness among these groups during the COVID-19 pandemic are lacking. Using data from the Survey of Health, Ageing and Retirement in Europe (SHARE) collected before (October 2019–March 2020) and during the pandemic (June–August 2020), we examine two loneliness outcomes: (1) “have you felt lonely recently?” (both datasets) and (2) “have you felt lonelier than before the pandemic?” (2020), and examine differences by partnership and parenthood status. Before COVID-19, those who lacked one tie but had the other (unpartnered parents or partnered childless) were at highest loneliness risk. During COVID-19, unpartnered and childless—especially unpartnered—remain at higher risk for loneliness, entering loneliness, and not “exiting” loneliness. We discuss these findings in light of family norms and needs in pandemic and non-pandemic times and provide recommendations for future research.

**Supplementary Information:**

The online version contains supplementary material available at 10.1007/s10433-022-00718-x.

## Introduction

Since the decade preceding the COVID-19 pandemic, scholars and public health officials have been increasingly concerned about loneliness among older adults, potentially driven by shifts such as decreasing fertility, the increasing prevalence of living alone, and other factors, especially in the United States and Europe (Aartsen and Jylha 2011; Dahlberg et al. (2022); Holt-Lunstad 2017; Holt-Lunstad et al. 2015; Klinenberg 2016; National Academies of Sciences, Engineering, and Medicine 2020; Verdery et al. 2019; Zoutewelle-Terovan & Liefbroer 2018). While loneliness appears to be increasing among the general population globally, older adults may be at heightened risk for loneliness and its consequences (Holt-Lunstad et al. 2015; Klinenberg 2016; Verdery et al. 2019), including poor physical health (Valdorta et al. 2016) and mortality (Holt-Lunstad et al. 2015). This is particularly a concern for older adults who do not have traditional family ties—like partners and children (Greenfield and Russell 2011; Hazer and Boylu 2010; Fokkema et al. 2012; Margolis et al. 2021; Zhang and Hayward 2001).

However, after the arrival of the COVID-19 pandemic, “physical distancing” was emphasized at national, regional, and local levels to mitigate the spread of the disease. These mitigation recommendations included avoidance of public social spaces, minimizing physical contact with others, “stay-at-home” orders, and full physical isolation of high-risk individuals, such as some older adults (Plümper and Neumayer 2020). While these mitigation efforts were likely useful to “slow the spread” of COVID-19 and reduce mortality, they may have exacerbated the risks of loneliness especially among older adults (Dahlberg 2021). This trend may have been particularly problematic for older adults with fewer family ties—such as unpartnered or childless older adults. Although there is a recent explosion of studies examining loneliness during COVID-19, with several studies focused on older adults (e.g., Bundy et al. 2021; Choi et al. 2021; Macdonald & Hulur 2021; Van Tilburg et al. 2021), no study to date has specifically examined the loneliness risk of unpartnered and childless older adults during COVID-19.

In this paper, we investigate if unpartnered and childless older adults report higher loneliness compared to their partnered and parent counterparts, respectively, and whether the gaps in loneliness between those with and those without these family ties have changed during the COVID-19 pandemic (compared to before). To do so, we analyze a representative sample of adults aged 50 + from the Survey of Health, Ageing and Retirement in Europe (SHARE), which includes data collected before the pandemic that was interrupted by COVID-19 (SHARE regular wave 8, October 2019–March 2020) as well as a special dataset with information collected during the pandemic (SHARE Corona Survey 1 (SCS1), June–August 2020). Specifically, we compare loneliness levels before and during the COVID-19 pandemic (regular wave 8 and SCS1) and examine older adults’ subjective assessments about whether their loneliness increased during the pandemic (SCS1).

## Loneliness

Loneliness is defined as a negative feeling that arises from discrepancies in individuals’ desired and actual social interactions and emotional support derived from these social contacts (Perlman & Peplau 1981; Tesch-Roemer & Huxhold 2019). Conceptually, loneliness is sometimes conflated with concepts of social isolation. Although having fewer social contacts is a risk factor for loneliness (Aartsen and Jylha 2011; Conkova et al. 2019), not all individuals who have lower social interaction feel lonely. For example, individuals with a higher number of social contacts may hold high expectations for social interaction frequency and may feel disappointed if their expectations are not met, thereby increasing risk of loneliness (Dykstra 2009). In this way, loneliness carries a subjective component that can vary according to individual and cultural expectations as well as family or lifestyle decisions. During 2020, loneliness may also be shaped by “physical distancing” behaviors that individuals adopted during the COVID-19 pandemic. Feelings of loneliness during COVID-19 may also differ depending on social network structures, such as availability of family ties, yielding different loneliness experiences for unpartnered and childless older adults.

## Older adults’ loneliness during COVID-19

Despite the elevated risk of isolation for older people during the COVID-19 pandemic, studies suggest that older adults were more resilient than younger adults to loneliness during COVID-19. Several online and nationally representative studies from multiple countries found that older age was a protective factor against loneliness (Beam and Kim 2020; Bu et al. 2020; Groarke et al. 2020; Li and Wang 2020; Luchetti et al. 2020; McQuaid et al. 2021; O’Sullivan et al. 2021; Palgi et al. 2020; Varga et al. 2021). These patterns may reflect a combination of factors, including older adults’ life course experiences (Carr 2020) and perhaps lower expectations for social interaction during COVID-19 (Dahlberg 2021).

Although older adults may have an advantage relative to younger adults, it is unclear whether older adults across the globe experienced increases in loneliness during COVID-19. For example, Choi et al. (2021) found that US older adults who limited their social interactions during the early stages of the pandemic (April to May 2020) were at higher risk for loneliness. Similar results based on SHARE data have been found among older Europeans during the summer of 2020 (Cohn-Schwartz et al. 2021; Reine 2021). On the other hand, in a nationally representative study with data from the USA, Luchetti and colleagues (2020) found no sustained increase in loneliness among older Americans. Furthermore, a qualitative US study found that older people who had lived with persistent loneliness before the pandemic did not report feeling lonelier than before COVID-19 (Bundy et al. 2021). Killgore et al. (2020) and Sutton et al. (2020) debated, via letters to the editor, the conflicting findings in online samples regarding American older adults’ experienced loneliness increases during COVID-19. Beyond the USA, there is evidence of increased loneliness among older adults in Switzerland (Macdonald & Hulur 2021), the Netherlands (Van Tilburg et al. 2021), and Hong Kong (Tso and Park 2020), but evidence of resilience to loneliness in Israel (Palgi et al. 2020).

Overall, existing studies on COVID-19 and older adults’ loneliness cross-nationally are difficult to compare due to wide variation in study designs in terms of sampling strategies and social contexts (e.g., local, regional, country variation in policy and COVID-19 mitigation strategies). Many studies conducted during the early phase of the COVID-19 pandemic were based on convenience samples, online only sampling techniques, etc. As Dahlberg (2021) argues, more work is needed using large, nationally representative samples of older adults.

## Loneliness among unpartnered and childless older adults

Research prior to the COVID-19 outbreak identifies partners and children as important family resources to buffer older adults’ loneliness. Being unpartnered, childless, or both are risk factors for loneliness among older adults (Dahlberg et al. 2022); Greenfield and Russell 2011; Hazer and Boylu 2010; Fokkema et al. 2012; Korepeckyj-Cox 1998; Margolis et al. 2021; Zhang & Hayward 2001). Therefore, unpartnered and childless older adults may also experience higher risk of loneliness during COVID-19. For example, unpartnered and childless older adults typically have broader networks of extended kin and non-kin ties (Djundeva et al. 2019; Mair 2019), which may have been more vulnerable to disruption during COVID-19 compared to partner and child ties. This network disruption during COVID-19 may have further enhanced loneliness risk among childless and unpartnered older adults.

On the other hand, linkages between parenthood, partnership, and loneliness may be particularly complex during COVID-19, and therefore, the associations between these factors may be complex. For example, older parents who are accustomed to frequent in-person contact with their children may experience an uptick in loneliness when that contact is disrupted. If this occurs, the pre-pandemic loneliness gap between older adults with and without children may narrow.

The few studies that examined changes in contacts with children during the pandemic found mixed results. Cohn-Schwartz and colleagues (2021) found that fewer contacts with children (and non-kin) were associated with feeling lonelier during the pandemic. However, Van Tilburg et al. (2021) found that older adults in the Netherlands who already had low contact with children and unmet social support needs had increased loneliness. Yet, declines in contact were not associated with increases in loneliness. The authors note that these findings could reflect different coping strategies against loneliness (Rokach & Brock 1998; Schoenmakers et al. 2012). For example, older adults might have temporarily lowered their expectations of interaction with family or increased digital contact with family (Arpino et al. 2021a; Dahlberg 2021; Freedman et al. 2021; Pan et al. 2021). More work is needed to understand older adults’ changes in loneliness and family support during COVID-19.

Overall, unpartnered older adults, childless older adults, and older adults who are both unpartnered and childless appear to be at higher risk of loneliness (compared to partnered, parents, and partnered parents) prior to COVID-19 and may remain so during COVID-19. However, COVID-19 mitigation efforts yielded many complex social disruptions for older adults of all family structures. Older adults likely adjusted their expectations of social contact and their methods of achieving it. Despite public health concerns about risks of loneliness among older adults before and during COVID-19, we were unable to find any existing studies that explore this topic using nationally representative data and that focus on the potential heightened loneliness risk for unpartnered and childless older adults.

## Research Questions

In this paper, we examine loneliness among older adults during the COVID-19 pandemic and examine if unpartnered and childless older adults are at heightened risk for loneliness. Specifically, we ask:

(RQ1): Did unpartnered and childless older adults report more loneliness than partnered and parents before the pandemic?
(RQ2): If so, have the gaps in loneliness between those with and those without these family ties changed?
(RQ3): Do unpartnered and childless older adults subjectively report feeling lonelier than before the start of the pandemic?
(RQ4): Are partnership and parenthood associated with the likelihood of starting, continuing or stopping to feel lonely after the onset of the pandemic?

## Methods

### Data

We use data from the Survey of Health, Ageing and Retirement in Europe (SHARE), a panel survey representative of the non-institutionalized population aged 50 + administered every 2 years since 2004 in several European countries and Israel (Börsch-Supan et al. 2013). We use data from wave 8, which started in October 2019 but was suspended in all countries in March 2020 due to the COVID-19 outbreak. Regular data collection is based on computer-assisted personal interviewing (CAPI), which provides pre-COVID information (“regular wave 8” hereinafter) (Börsch-Supan 2020). A special dataset, SHARE Corona Survey 1 (Börsch-Supan 2022), was added to wave 8. This survey has been administered with CATI (computer-assisted telephone interviewing) between June and August 2020 to collect information on individuals’ behaviors and conditions during the pandemic (SHARE Corona Survey 1; SCS1 hereinafter). The later wave provides information collected after the onset of the pandemic (we will refer to it as “during pandemic” data). We excluded observations from Portugal (because Portugal started the fieldwork of the regular wave 8 only a few weeks before the start of the first lockdown due to the pandemic), thus restricting the analyses to individuals from the 27 countries included in both regular and SCS1 data. After discarding relatively few observations with missing values (1941 from regular wave 8 (4.2%) and 2,059 from SCS1 (3.7%), our analytic samples comprised 44,329 (regular wave) and 53,820 (SCS1) individuals. 35,068 individuals (70,136 observations) are included in both datasets. Data from Austria have been removed in a robustness check due to different timing of their SCS1 data collection (July–September).

### Dependent variables

We examine two single-item outcomes of loneliness (feeling lonely and feeling more lonely during the pandemic). Feelings of loneliness (“How much of the time do you feel lonely?”) were assessed before (regular wave 8, 2019–2020) and during the pandemic (SCS1, 2020) and are used to answer RQ1 and RQ2. Given only 7 percent of the sample felt lonely “often,” we combine “often” and “some of the time” (1 = felt lonely often/some of the time; 0 = felt lonely hardly ever or never). Combining these two categories is also practical because the group who felt lonely “often” or “some of the time” received an additional follow-up question during the pandemic (SCS1), which we describe below for use as our second outcome measure.

The second indicator of loneliness was asked only once during the pandemic (SCS1, 2020) and assessed if respondents felt more lonely during the pandemic as a follow-up to the standard feeling lonely question described above. In SCS1 (2020), respondents who indicated that they felt lonely “often” or “some of the time” were additionally asked “Has that been more so, less so, or about the same as before the outbreak of Corona?”. We also dichotomized this measure (1 = felt more lonely during the pandemic, 0 = felt the same or less lonely during the pandemic, or did not indicate they were lonely in the general question and so received a 0 on “felt more lonely” for this second outcome). This was used to answer RQ3.

To observe potential changes in loneliness with more detail (RQ4), by comparing the values of the first outcome variable before and during the pandemic, we also created a third outcome variable which is a multicategory variable to track four possible outcomes from 2019–2020 to 2020 (not lonely both before and during the pandemic, lonely both before and during the pandemic, from not lonely to lonely, and from lonely to not lonely). We did not account here for the values of the second outcome variable. Thus, if a person answered “often” to the questions about feeling of loneliness both before and during the pandemic, this person is coded as “lonely both before and during the pandemic,” independently of whether this has been more or less compared to before the pandemic.

### Explanatory and control variables

Our primary explanatory variables are partnership and parenthood status, measured with dummy variables indicating whether respondents are unpartnered (vs being in a partnership) and childless (vs having at least one child—biological, adopted, or step).

Socio-demographic controls include sex (reference = male), age (dummy variables for 10-year categories; reference = 50–59); education (reference = low), employment status (working; retired—reference; other, including unemployed, permanently sick or disabled, homemaker), country of residence (reference = Italy). Educational groups are defined based on the International Standard Classification of Education (ISCED; http://www.uis.unesco.org/): low (ISCED 0–1, no or primary education, and ISCED 2, lower secondary education—reference), medium (ISCED 3–4, higher secondary education), and high (ISCED 5–6, tertiary education). We also control for health status: self-reports of doctor-diagnosed conditions (hypertension, diabetes, cancer, lung disease, heart disease, stroke, and arthritis) with a dummy variable for at least one condition, a binary variable measuring global activity limitations (GALI), with a value of 1 for those whose activities are limited or severely limited because of health problems. In a robustness check, we also add a control for whether individuals other than the partner live in the household (versus not).

### Statistical analyses

First, we calculate descriptive statistics of all variables from before (regular wave 8, 2019–2020) and during the pandemic (SCS1, June and August 2020). We also present percentage breakdowns by country to illustrate the composition of our sample (Table 1).

**Table 1:** Descriptive statistics (%) on all variables separately for the regular wave 8 (before the pandemic) and SHARE Corona Survey 1 (during the pandemic) samples

Variable	Period		Variable	Period	
	Before	During		Before	During
Feel lonely	27.8	28.6	Netherlands	4.0	1.4
Felt lonelier than before pandemic		11.6	Spain	4.4	3.9
Childless	8.7	8.7	Italy	4.6	7.0
Unpartnered	28.9	28.4	France	5.4	3.9
Female	57.7	57.9	Denmark	4.8	3.7
*Age (mean)*	70.4	70.8	Greece	6.4	6.8
Age 50–59	12.4	10.7	Switzerland	4.2	3.5
Age 60–69	36.8	36.7	Belgium	4.4	7.1
Age 70–79	33.1	33.6	Israel	1.9	2.8
Age 80–89	15.6	16.4	Czech Republic	5.9	4.9
Age 90 +	2.1	2.5	Poland	4.4	5.4
*Education*			Luxembourg	2.0	1.6
Low	17.5	17.3	Hungary	1.7	1.9
Medium	17.0	17.4	Slovenia	5.3	5.7
High	65.5	65.3	Estonia	6.5	8.4
*Work status*			Croatia	2.6	3.8
Retired	69.6	64.6	Lithuania	3.0	2.2
Working	17.6	21.0	Bulgaria	1.9	1.5
Other	12.8	14.4	Cyprus	1.1	1.4
Illness	55.8	54.0	Finland	2.5	2.5
GALI	49.1	47.4	Latvia	1.6	1.8
*Country*			Malta	1.7	1.6
Austria	3.4	5.0	Romania	2.7	2.7
Germany	6.4	5.2	Slovakia	2.1	1.7
Sweden	5.2	2.5	n	44,329	53,820

Unweighted estimates. Data Source: SHARE wave 8 and SHARE Corona Survey 1, release version 8.0.0

Next, we estimate logistic regression models of our two binary outcome measures (outcomes 1 and 2). Given that our first outcome was asked twice (before and during the pandemic), we estimate three sets of models that include feeling lonely before the pandemic (regular wave 8, 2019–2020; RQ1), feeling lonely during the pandemic (SCS1, 2020; RQ2), and feeling that loneliness increased during the pandemic (SCS1, 2020 asked of the subset who felt lonely often/some of the time in SCS1; RQ3) (Table 2). For each of these, we include four models fully adjusted for control variables that incrementally add explanatory variables, including childlessness (Model 1, M1), unpartnered (Model 2, M2), both (Model 3, M3), both variables and their interaction (Model 4, M4). All models presented in Table 2 also include dummy variables for each country (not shown in tables).

**Table 2:** Estimated coefficients from logistic regressions

Independent variables	Before the pandemic (regular wave 8)				During the pandemic (SHARE Corona Survey 1)							
	Do you feel lonely?				Do you feel lonely?				Have felt lonelier than before the pandemic?			
	(M1)	(M2)	(M3)	(M4)	(M1)	(M2)	(M3)	(M4)	(M1)	(M2)	(M3)	(M4)
Childless	0.57***		0.28***	0.43***	0.51***		0.20***	0.21***	0.17***		0.00	-0.09
	(0.04)		(0.04)	(0.06)	(0.03)		(0.04)	(0.06)	(0.05)		(0.05)	(0.08)
Unpartnered		0.97***	0.93***	0.96***		0.94***	0.91***	0.91***		0.52***	0.52***	0.51***
		(0.02)	(0.03)	(0.03)		(0.02)	(0.02)	(0.02)		(0.03)	(0.03)	(0.03)
Childless * Unpartnered				-0.27***				-0.01				0.15
				(0.08)				(0.07)				(0.10)
Female	0.43***	0.26***	0.27***	0.26***	0.60***	0.42***	0.43***	0.43***	0.64***	0.54***	0.54***	0.54***
	(0.02)	(0.02)	(0.02)	(0.02)	(0.02)	(0.02)	(0.02)	(0.02)	(0.03)	(0.03)	(0.03)	(0.03)
Age (Ref.: 50–59)												
Age 60–69	0.03	-0.02	-0.01	-0.01	0.05	0.00	0.01	0.01	0.04	0.01	0.01	0.02
	(0.04)	(0.04)	(0.04)	(0.04)	(0.04)	(0.04)	(0.04)	(0.04)	(0.06)	(0.06)	(0.06)	(0.06)
Age 70–79	0.15***	0.05	0.06	0.06	0.22***	0.13***	0.14***	0.14***	0.15**	0.10	0.10	0.10
	(0.05)	(0.05)	(0.05)	(0.05)	(0.04)	(0.05)	(0.05)	(0.05)	(0.06)	(0.06)	(0.06)	(0.06)
age 80–89	0.59***	0.36***	0.38***	0.38***	0.63***	0.42***	0.44***	0.44***	0.43***	0.30***	0.30***	0.30***
	(0.05)	(0.05)	(0.05)	(0.05)	(0.05)	(0.05)	(0.05)	(0.05)	(0.07)	(0.07)	(0.07)	(0.07)
age 90 +	0.96***	0.59***	0.60***	0.60***	0.80***	0.44***	0.45***	0.45***	0.39***	0.17*	0.17*	0.17*
	(0.08)	(0.09)	(0.09)	(0.09)	(0.07)	(0.07)	(0.07)	(0.07)	(0.10)	(0.10)	(0.10)	(0.10)
Education (Ref.: low)												
Medium education	− 0.18***	**− **0.15***	**− **0.15***	**− **0.15***	**− **0.11***	**− **0.09***	**− **0.10***	**− **0.10***	0.03	0.04	0.04	0.04
	(0.04)	(0.04)	(0.04)	(0.04)	(0.03)	(0.04)	(0.04)	(0.04)	(0.05)	(0.05)	(0.05)	(0.05)
High education	**− **0.33***	**− **0.27***	**− **0.27***	**− **0.27***	**− **0.25***	**− **0.20***	**− **0.20***	**− **0.20***	**− **0.06	**− **0.03	**− **0.03	**− **0.03
	(0.03)	(0.03)	(0.03)	(0.03)	(0.03)	(0.03)	(0.03)	(0.03)	(0.04)	(0.04)	(0.04)	(0.04)
Work status (Ref.: retired)												
working	**− **0.20***	**− **0.23***	**− **0.23***	**− **0.23***	**− **0.22***	**− **0.23***	**− **0.23***	**− **0.23***	**− **0.25***	**− **0.26***	**− **0.26***	**− **0.26***
	(0.04)	(0.04)	(0.04)	(0.04)	(0.03)	(0.03)	(0.03)	(0.03)	(0.05)	(0.05)	(0.05)	(0.05)
Other	0.22***	0.22***	0.22***	0.22***	0.15***	0.16***	0.17***	0.17***	**− **0.02	**− **0.01	**− **0.01	**− **0.01
	(0.04)	(0.04)	(0.04)	(0.04)	(0.03)	(0.03)	(0.03)	(0.03)	(0.04)	(0.04)	(0.04)	(0.04)
Illness	0.09***	0.08***	0.08***	0.08***	0.20***	0.19***	0.19***	0.19***	0.21***	0.21***	0.21***	0.21***
	(0.03)	(0.03)	(0.03)	(0.03)	(0.02)	(0.03)	(0.03)	(0.03)	(0.03)	(0.03)	(0.03)	(0.03)
GALI	0.56***	0.57***	0.56***	0.56***	0.43***	0.43***	0.43***	0.43***	0.34***	0.33***	0.33***	0.33***
	(0.03)	(0.03)	(0.03)	(0.03)	(0.02)	(0.03)	(0.03)	(0.03)	(0.03)	(0.03)	(0.03)	(0.03)
Constant	**− **1.11***	**− **1.07***	**− **1.11***	**− **1.11***	**− **1.15***	**− **1.11***	**− **1.14***	**− **1.14***	**− **2.14***	**− **2.13***	**− **2.13***	**− **2.13***
	(0.07)	(0.07)	(0.07)	(0.07)	(0.06)	(0.06)	(0.06)	(0.06)	(0.08)	(0.08)	(0.08)	(0.08)
n	44,329	44,329	44,329	44,329	53,820	53,820	53,820	53,820	53,820	53,820	53,820	53,820

Unweighted estimates. This table includes full results from logistic regression models, except coefficients for country dummy variables. The four considered models are all fully adjusted for control variables and incrementally add explanatory variables: childlessness (Model 1, M1), unpartnered (Model 2, M2), both (Model 3, M3), both variables and their interaction (Model 4, M4). Standard errors in parentheses. *p < 0.1; **p < 0.05; ***p < 0.01

For ease and clarity of interpretation, and to avoid issues with comparison of coefficients from different logistic regressions, Table 3 includes calculated average marginal effects (AMEs) (Mood 2010). Due to the binary nature of our outcomes and explanatory variables, the AMEs are to be interpreted as the discrete effect of the variable, or the difference between the predicted probabilities (in percentage points; pp) across the groups being compared (e.g., childless vs parents). In model M4 that includes the interaction term, we calculated the AMEs of each explanatory variable (e.g., childlessness) separately for the categories of the other explanatory variable (unpartnered and partnered) to allow for clearer interpretation of the potential joint effect of being unpartnered and childless on each outcome at each time point. For the main analysis (Tables 2 and 3), all models are estimated separately according to the cross-sectional sample size of each wave (regular wave 8 and SCS1).

**Table 3:** Average marginal effects (AMEs) of being childless and of being unpartnered on the probability of feeling currently lonely or lonelier than before the pandemic

AME of:	Before the pandemic (regular wave 8)				During the pandemic (SHARE Corona Survey 1)							
	Do you feel lonely?				Do you feel lonely?				Have felt lonelier than before the pandemic?			
	(M1)	(M2)	(M3)	(M4)	(M1)	(M2)	(M3)	(M4)	(M1)	(M2)	(M3)	(M4)
Childless	0.1126***		0.0504***	0.1005***		0.0377***	0.0177***		0.0004			
	(0.0078)		(0.0073)		(0.0071)		(0.0067)		(0.0050)		(0.0046)	
Unpartnered		0.1874***	0.1794***		0.1857***	0.1795***		0.0545***	0.0544***			
		(0.0050)	(0.0051)			(0.0046)	(0.0047)			(0.0034)	(0.0034)	
Childless												
Partnered				0.0758***				0.0367***				-0.0077
				(0.0112)				(0.0099)				(0.0065)
Unpartnered				0.0367***				0.0444***				0.0074
				(0.0111)				(0.0102)				(0.0074)
Unpartnered												
Parents				0.1843***				0.1789***				0.0526***
				(0.0054)				(0.0050)				(0.0037)
Childless				0.1451***				0.1866***				0.0677***
				(0.0148)				(0.0133)				(0.0092)
n	44,329	44,329	44,329	44,329	53,820	53,820	53,820	53,820	53,820	53,820	53,820	53,820

Unweighted estimates. The AMEs reported in this table have been calculated based on estimates from logistic regression models reported in Table 2. Control variables have been averaged out. The four considered models are all fully adjusted for control variables and incrementally add explanatory variables: childlessness (Model 1, M1), unpartnered (Model 2, M2), both (Model 3, M3), both variables and their interaction (Model 4, M4). Standard errors in parentheses. *p < 0.1; **p < 0.05; ***p < 0.01

Finally, for our third multicategory outcome we estimated multinomial logistic regression models (RQ4). To ease the interpretation of the signs of the associations, in Table 4 we report coefficients estimated from two statistically equivalent models that only differ for the reference category of the outcome. First, we report estimated coefficients for being (1) “*lonely both before and during the pandemic*” and (2) “*from not lonely to lonely*” with both compared to “*not lonely both before and during the pandemic*.” In this case, we assess the associations between our explanatory variables with the probability of remaining lonely in both waves or of “entering” loneliness compared to not being lonely in both waves. Next, we provide coefficients for going (3) “*from lonely to not lonely*” compared to the reference category of being “*lonely both before and during the pandemic*”, as this represented a more meaningful contrast. These results inform us about the association of our explanatory variables with the probability of “exiting” loneliness compared to being lonely both before and during the pandemic. Because this portion of the analysis seeks to compare changes from one wave to the other, we limit this sample to respondents who answered the loneliness questions in both the regular wave 8 and the SCS1 (35,068 individuals, or 79% of the regular wave sample).

**Table 4:** Modeling changes in loneliness: Estimated coefficients from multinomial logistic regressions for the explanatory variables selecting only individuals included in both the regular wave 8 and SHARE Corona Survey 1data

Independent variables	(M3)	(M4)
*(1) Continued loneliness. Effect on: Lonely both before and during the pandemic (ref.* = *not lonely both before and during the pandemic)*		
Childless	0.34***	0.52***
	(0.05)	(0.09)
unpartnered	1.37***	1.40***
	(0.04)	(0.04)
childless * unpartnered		**− **0.28**
		(0.11)
*(2) Entering loneliness. Effect on: From not lonely to lonely (ref.* = *not lonely both before and during the pandemic)*		
childless	0.10	0.02
	(0.06)	(0.10)
unpartnered	0.67***	0.66***
	(0.04)	(0.04)
childless * unpartnered		0.12
		(0.13)
*(3) Exiting loneliness. Effect on: From lonely to not lonely (ref.* = *lonely both before and during the pandemic)*		
childless	**− **0.17**	**− **0.24**
	(0.07)	(0.11)
unpartnered	**− **0.61***	**− **0.62***
	(0.05)	(0.05)
childless * unpartnered		0.07
		(0.14)
n	35,068	35,068

Unweighted estimates. The outcome has four categories that compare the pre-COVID and during-COVID lonely feelings. Estimates from two (statistically equivalent models) are presented that differ for the reference category. We present coefficients from the first model where the reference category is “*not lonely both before and during the pandemic*” for the first two categories of the outcome, while for the last one (*from lonely to not lonely*) we present results from the second model where the reference category was “*lonely both before and during the pandemic.*” All independent variables are measured at the time of the regular wave. The four considered models are all fully adjusted for control variables and incrementally add explanatory variables: childlessness (Model 1, M1), unpartnered (Model 2, M2), both (Model 3, M3), both variables and their interaction (Model 4, M4). Here, results for M3 and M4 only are displayed for simplicity

## Results

Descriptively, there is very little difference between the independent and control variables before (2019–2020) and during (2020) the pandemic (Table 1). In the outcome variables, the prevalence of feeling lonely only increased by 0.8 percentage points during (2020) compared to before (2019–2020) the pandemic. Yet, 11.6% perceived themselves as more lonely during (2020).

## Multivariable results

### Loneliness before vs during the pandemic

Table 2 reports estimated coefficients of the explanatory variables from the logistic regression models described above, and Table 3 displays the corresponding AMEs. Table 2 shows that before the pandemic (regular wave 8), childless and unpartnered individuals were at higher risk of loneliness compared to those with these family ties (RQ1). The positive and statistically significant (*p* < 0.01) associations remain in M3 where the two variables are included together. However, the coefficient of childless is considerably reduced. Likewise, the AME of childlessness reduces from 11.3 pp to 5 pp (Table 3). Instead, the AME of unpartnered remains fairly stable in M3. Individuals who lack a partner are 18 pp more likely to feel lonely as compared to their partnered counterparts, which is a rather strong association. Due to a correlation between being childless and unpartnered, in M1 the effect of childlessness is partially confounded with being unpartnered. When the two effects are separated, however, the association between childlessness and loneliness is reduced. The interaction between childless and unpartnered is negative and statistically significant (M4, Table 2; p < 0.01). Consequently, the loneliness effect of lacking one tie is stronger when the other tie is present. For example, the AME of childlessness is 7.6 for partnered and 3.7 for unpartnered (Table 3). Similarly, the AME of being unpartnered is higher among parents (18.4 pp) than childless (14.5 pp) individuals. These differences are statistically significant (*p* < 0.01) and substantial in relative terms especially for childlessness.

During the pandemic (SCS1), overall, associations between parenthood and partnership status and feeling lonely barely changed (RQ2). The AMEs for childlessness and being unpartnered on loneliness during the pandemic are similar to those observed before: 3.8 pp (vs 5 pp) for childless and 18 pp (vs 17.9) for unpartnered (M3, Table 3). The differences between the associations during versus before the pandemic were also not statistically significant (*p* > 0.1). This was tested by pooling the regular and SCS1samples and adding a dummy variable for the SCS1sample interacted with the explanatory variables. None of the interactions (separately or jointly) were statistically significant, confirming the stability of associations across the two periods as shown by the separate analyses in model M3 of Tables 2 and 3.

We conducted similar tests for the other models and interactions. The only one that resulted to be statistically significant (*p* < 0.05) referred to the test of the triple interaction between childlessness, being unpartnered, and the SCS1sample dummy. This interaction reveals that before the pandemic, there was a statistically significant interaction between being childless and being unpartnered (where lacking one tie had a stronger effect when the other tie was present), but that interaction is not present during the pandemic (M4, Table 2). As a result, the AMEs of being unpartnered (childless or not) are very similar during the pandemic, *and* the AMEs for childless (partnered or not) are also very similar during the pandemic. In other words, prior to the pandemic—lacking one tie was more strongly associated with loneliness when the other tie was present. During the pandemic, these effects “uncouple” and are associated with loneliness more independently.

### Feeling as though loneliness increased during the pandemic

Our second outcome examines whether people subjectively assessed that they felt more lonely during the pandemic (last four columns of Tables 2 and 3; RQ3). In this case, only being unpartnered displays a positive and statistically significant coefficient and AME. Being unpartnered during the pandemic was associated with 5.4 pp higher probability of feeling lonelier than before the outbreak as compared with individuals in a partnership (M3 in Table 3). Although this association is slightly stronger among childless (6.7 pp) than among parents (5.3 pp; M4 in Table 3), the difference between the AME of being unpartnered does not vary significantly (*p* > 0.1) by parenthood status. This is also reflected in the insignificant coefficient of the interaction between being unpartnered and childlessness (M4, Table 2).

### Changes and stability in feeling lonely compared to before the pandemic

Our third outcome (having felt lonely before and/or since the start of the pandemic) was used to test whether childlessness or being unpartnered was associated with changes in the feeling of loneliness since the start of the pandemic (RQ4; Table 4). Consistent with results shown in Tables 2 and 3, the model without the interaction (M3) confirms that childless and unpartnered individuals were more likely to report feelings of loneliness both before and during the pandemic compared to their partnered and parent counterparts (Table 4, Part 1). Unpartnered were more likely to “become lonely” during the pandemic (compared to not reporting this feeling in both periods), unlike childless (Table 4, Part 2). Among those who felt lonely before the pandemic, childless and unpartnered were less likely to “exit” loneliness (Table 4, Part 3). The interaction terms between childlessness and partnership (M4) suggest that lacking one tie does not influence the probability of changes in loneliness due to the other (consistent with Tables 2 and 3 for the second outcome).

### Robustness checks

We performed a series of robustness checks on respondents’ household composition and unpartnered marital status. In Table S.1 of Supplementary Materials, we first replicate the results of Table 2 adding a control variable for “others in the household” to determine if having a person other than the respondent or the partner changes the patterns we observed. Results barely changed adding this additional control variable. Second, in order to better determine the relationship between being unpartnered and loneliness, we separate the “unpartnered” category into never married, separated/divorced, and widowed. All three types of unpartnered individuals are at higher risk of loneliness both before and during the pandemic with widowed being at highest risk. Additionally, in the before-COVID period the negative and statistically significant interactions between being unpartnered and childlessness found in Table 2 are confirmed for separated/divorced and never married older adults but not for widowed. Therefore, the effect of childlessness on loneliness is similar for widowed and married individuals (and higher than that for separated/divorced and never married). We also find a positive interaction between childlessness and widowhood for the second outcome, suggesting that the effect of childlessness on the probability of having felt lonelier than before the pandemic is higher for widowed than for married (and all the other partnership groups; for these the coefficients of the interactions are small and not significant). More precisely, a statistically significant effect of childlessness is only found for widowed.

Finally, we conducted additional miscellaneous robustness checks that excluded: (1) respondents who tested positive for COVID-19 and those who had a close relative or friend who tested positive or died during the pandemic time; (2) respondents from Austria due to data timing differences; and 3) respondents who were not observed in both surveys. These final miscellaneous robustness checks indicated no variation in the patterns observed (see Table S.2 of Supplementary Materials).

## Discussion

This paper examines loneliness among older adults in Europe and Israel before and during the COVID-19 pandemic, with a particular emphasis on loneliness by partnership and parenthood statuses. We found that prior to the pandemic, unpartnered parents had the highest risk of loneliness, followed by partnered childless adults, but “kinless” older adults (unpartnered and childless) were not lonelier than these other two groups who lacked only one tie. Additionally, we found that being unpartnered during the pandemic was associated with a higher likelihood of becoming lonely. Finally, being unpartnered and childless were associated with a higher likelihood of staying lonely during COVID-19. Below, we discuss the meaning of these findings, as well as potential explanations, limitations, and future avenues for research.

### Pre-pandemic loneliness risk for unpartnered parents, partnered childless

Our first research question asked whether unpartnered and childless older adults were at higher risk of feeling lonely than partnered and parents before the pandemic (RQ1). Prior to the pandemic, both unpartnered and childless older adults appear to be at higher risk for loneliness compared to their partnered and parental counterparts. Consistent with prior research, being unpartnered is a stronger predictor of loneliness than being childless (Margolis et al. 2021; Zhang and Hayward 2001). However, risk of loneliness is not compounded (or higher) for older adults who are “kinless” (unpartnered and childless at the same time) as compared to being an unpartnered parent. Our findings suggest that lacking one family tie but having the other tie places older adults at higher risk of loneliness prior to the pandemic than someone who is “kinless,” with unpartnered parents having the highest probability of pre-pandemic loneliness. Although there could be a number of possible explanations for this finding, this pattern could reflect expectations and belief in “traditional” familism values. Older adults who maintain at least one of the “traditional” family ties may be more likely to expect connections from family, and therefore more likely to feel a sense of loneliness if they lack one of the other ties. For unpartnered parents, in particular, the presence of children without a partnership may contribute to loneliness as they are not embedded in a “traditional” family status. One of our robustness checks showed that this is especially the case for older adults who never married and those who lost their partner because of divorce/separation.

### Increased loneliness during the pandemic, especially for unpartnered and childless

In answer to our second research question, we found that during the pandemic, the loneliness gaps by partnership and parenthood status did not change substantially, but the interaction between the two that was found before the pandemic was not confirmed. In addition, unpartnered older adults were more likely to increase their levels of loneliness (RQ3), become lonely if they were not already, and not “exit” loneliness if they were already lonely (RQ4). Childless older adults were more likely to be lonely and not “exit” loneliness, but they were not more likely to become lonely if they were not already. So-called kinless older adults (unpartnered childless) were not necessarily at higher risk of loneliness during the pandemic than each group separately. This finding may reflect and support the resilience of “already lonely” older adults (Bundy et al. 2021) such that “kinless” older adults potentially have long-standing resources and preparations (psychological, emotional, and instrumental) to cope with limited social resources and thus manage loneliness due to the absence of these traditional family resources. Additionally, “kinless” older adults, whose networks are more friend-based (Mair 2019), may have reduced their expectations for in-person social contacts thereby reducing their discrepancy in desired versus actual interactions and subsequent likelihood of becoming lonely during the pandemic (Dahlberg 2021). Nevertheless, our findings underscore prior research on the salience of lacking particularly a partner in terms of loneliness among older adults (Greenfield and Russell 2011) and hint to the heightened isolation experienced by the unpartnered during COVID-19 mitigation efforts.

### Limitations, future research and conclusions

Our results provide several avenues for future research. First, our analysis considers two measures of loneliness that show variation, but future studies should examine additional well-being outcomes, such as anxiety and depression, among childless and unpartnered older adults during the pandemic. Second, similar to studies on the effects of social contacts on depression during the pandemic (Arpino et al. 2021b; Litwin and Levinsky 2021), future work should explore in-depth information about social interactions (e.g., care and help received, social contacts including digital ones) that may mitigate potential social isolation. Third, further research can investigate whether the pandemic levels or reinforces gender differences in loneliness by parenthood and partnership status identified in prior research (Greenfield and Russell 2011; Zhang and Hayward 2001). Finally, cross-national heterogeneity in loneliness was evident before the pandemic (Fokkema et al. 2012; Nyqvist et al. 2019; Rapolienė and Aartsen 2021) and future studies should examine cross-country differences in the changes in loneliness during the pandemic (as done by Atzendorf and Gruber 2021) among childless and unpartnered older individuals.

Despite these limitations, our results highlight important conceptual considerations about loneliness among childless, and particularly unpartnered, older adults. These groups are at higher risk regardless of global events and felt lonelier during the pandemic. While “physical distancing,” “lockdown,” and “stay-at-home” mitigation measures provided protection, the restrictions on face-to-face interactions and public spaces for socializing also removed key sources of social integration for those who are already more likely to spend time alone (e.g., unpartnered). As the population of unpartnered and childless older adults grows globally, future public health strategies should seek a balanced mitigation approach that also considers the consequences of isolation, particularly for populations who are already at higher risk for loneliness.

## Supplementary Information

Below is the link to the electronic supplementary material.

Supplementary file1 (DOCX 19 kb)
