# Supplementary material for: Loneliness before and during the COVID-19 pandemic—are unpartnered and childless older adults at higher risk?
Source: Eur J Ageing. 2022 Jul 19;19(4):1327–38. doi: 10.1007/s10433-022-00718-x (PMC9294803; doi:10.1007/s10433-022-00718-x)
Supplement: Supplementary file 1 — Supplementary file1 (DOCX 19 kb) [file 10433_2022_718_MOESM1_ESM.docx]

**Supplementary Materials**

**Table S.1 – Robustness checks of the main analyses presented in Table 2 above. Estimated coefficients for the explanatory variables from logistic regressions**

| Independent variables | Before the pandemic (regular wave 8) | | | | During the pandemic (SHARE Corona Survey 1) | | | | | | | |
| --- | --- | --- | --- | --- | --- | --- | --- | --- | --- | --- | --- | --- |
|  | Do you feel lonely? | | | | Do you feel lonely? | | | | Have felt lonelier than before the pandemic? | | | |
|  | (M1) | (M2) | (M3) | (M4) | (M1) | (M2) | (M3) | (M4) | (M1) | (M2) | (M3) | (M4) |
| 1) *Controlling for whether individuals other than the respondent and his/her partner are present in the household* | | | | | | | | | | | | |
| childless | 0.50*** |  | 0.24*** | 0.38*** | 0.42*** |  | 0.16*** | 0.15*** | 0.11** |  | -0.03 | -0.09 |
|  | (0.04) |  | (0.04) | (0.06) | (0.03) |  | (0.04) | (0.06) | (0.05) |  | (0.05) | (0.08) |
| unpartnered |  | 0.92*** | 0.89*** | 0.92*** |  | 0.89*** | 0.86*** | 0.86*** |  | 0.47*** | 0.48*** | 0.46*** |
|  |  | (0.03) | (0.03) | (0.03) |  | (0.02) | (0.02) | (0.02) |  | (0.03) | (0.03) | (0.03) |
| childless * unpartnered |  |  |  | -0.23*** |  |  |  | 0.03 |  |  |  | 0.18 |
|  |  |  |  | (0.08) |  |  |  | (0.07) |  |  |  | (0.11) |
| others in the household | -0.55*** | -0.39*** | -0.37*** | -0.36*** | -0.59*** | -0.43*** | -0.42*** | -0.42*** | -0.50*** | -0.40*** | -0.40*** | -0.40*** |
|  | (0.04) | (0.04) | (0.04) | (0.04) | (0.03) | (0.03) | (0.03) | (0.03) | (0.05) | (0.05) | (0.05) | (0.05) |
| 2) *Distinguishing between never married, separated/divorced and widowed* | | | | | | | | | | | | |
| childless | 0.57*** |  | 0.35*** | 0.43*** | 0.51*** |  | 0.24*** | 0.21*** | 0.17*** |  | -0.02 | -0.09 |
|  | (0.04) |  | (0.04) | (0.06) | (0.03) |  | (0.04) | (0.06) | (0.05) |  | (0.05) | (0.08) |
| never married |  | 0.96*** | 0.74*** | 0.86*** |  | 0.98*** | 0.84*** | 0.82*** |  | 0.56*** | 0.57*** | 0.60*** |
|  |  | (0.05) | (0.06) | (0.08) |  | (0.04) | (0.05) | (0.07) |  | (0.06) | (0.07) | (0.10) |
| separated/divorced |  | 0.84*** | 0.83*** | 0.85*** |  | 0.86*** | 0.85*** | 0.85*** |  | 0.59*** | 0.60*** | 0.60*** |
|  |  | (0.04) | (0.04) | (0.04) |  | (0.04) | (0.04) | (0.04) |  | (0.05) | (0.05) | (0.05) |
| widowed |  | 1.05*** | 1.05*** | 1.05*** |  | 0.98*** | 0.97*** | 0.97*** |  | 0.47*** | 0.47*** | 0.45*** |
|  |  | (0.03) | (0.03) | (0.03) |  | (0.03) | (0.03) | (0.03) |  | (0.04) | (0.04) | (0.04) |
| childless * never married |  |  |  | -0.25** |  |  |  | 0.04 |  |  |  | 0.03 |
|  |  |  |  | (0.12) |  |  |  | (0.10) |  |  |  | (0.14) |
| childless * separated/divorced |  |  |  | -0.23* |  |  |  | 0.04 |  |  |  | 0.03 |
|  |  |  |  | (0.14) |  |  |  | (0.13) |  |  |  | (0.18) |
| childless * widowed |  |  |  | -0.04 |  |  |  | 0.06 |  |  |  | 0.30** |
|  |  |  |  | (0.12) |  |  |  | (0.11) |  |  |  | (0.14) |
| n | 44329 | 44329 | 44329 | 44329 | 53820 | 53820 | 53820 | 53820 | 53820 | 53820 | 53820 | 53820 |
| Note: Unweighted estimates. All control variables described in the main documents are included in all models. The four considered models are all fully adjusted for control variables and incrementally add explanatory variables: childlessness (Model 1, M1), unpartnered (Model 2, M2), both (Model 3, M3), both variables and their interaction (Model 4, M4). Standard errors in parentheses. * p < 0.1; ** p < 0.05; *** p < 0.01. | | | | | | | | | | | | |

**Table S.2 – Additional robustness checks of the main analyses presented in Table 2. Estimated coefficients for the explanatory variables from logistic regressions**

| Independent variables | Before the pandemic (regular wave 8) | | | | During the pandemic (SHARE Corona Survey 1) | | | | | | | |
| --- | --- | --- | --- | --- | --- | --- | --- | --- | --- | --- | --- | --- |
|  | Do you feel lonely? | | | | Do you feel lonely? | | | | Have felt lonelier than before the pandemic? | | | |
|  | (M1) | (M2) | (M3) | (M4) | (M1) | (M2) | (M3) | (M4) | (M1) | (M2) | (M3) | (M4) |
| 1) *Excluding respondents who tested positive and those who had some close relative of friend who tested positive and/or died (during the pandemic only)* | | | | | | | | | | | | |
| childless |  |  |  |  | 0.52*** |  | 0.22*** | 0.22*** | 0.19*** |  | 0.03 | -0.09 |
|  |  |  |  |  | (0.03) |  | (0.04) | (0.06) | (0.05) |  | (0.05) | (0.08) |
| unpartnered |  |  |  |  |  | 0.94*** | 0.91*** | 0.91*** |  | 0.52*** | 0.51*** | 0.49*** |
|  |  |  |  |  |  | (0.02) | (0.02) | (0.02) |  | (0.03) | (0.03) | (0.03) |
| childless * unpartnered |  |  |  |  |  |  |  | -0.00 |  |  |  | 0.18 |
|  |  |  |  |  |  |  |  | (0.07) |  |  |  | (0.11) |
| n |  |  |  |  | 51153 | 51153 | 51153 | 51153 | 51153 | 51153 | 51153 | 51153 |
| 2) *Excluding respondents from Austria because of different timing of the covid survey compared to the other countries (during the pandemic only)* | | | | | | | | | | | | |
| childless |  |  |  |  | 0.53*** |  | 0.22*** | 0.25*** | 0.18*** |  | 0.02 | -0.06 |
|  |  |  |  |  | (0.04) |  | (0.04) | (0.06) | (0.05) |  | (0.05) | (0.08) |
| unpartnered |  |  |  |  |  | 0.94*** | 0.91*** | 0.92*** |  | 0.51*** | 0.51*** | 0.50*** |
|  |  |  |  |  |  | (0.02) | (0.02) | (0.03) |  | (0.03) | (0.03) | (0.03) |
| childless * unpartnered |  |  |  |  |  |  |  | -0.04 |  |  |  | 0.13 |
|  |  |  |  |  |  |  |  | (0.07) |  |  |  | (0.10) |
| n |  |  |  |  | 49191 | 49191 | 49191 | 49191 | 49191 | 49191 | 49191 | 49191 |
| 3) *Selecting only individuals observed both in the regular and covid surveys* | | | | | | | | | | | | |
| childless | 0.57*** |  | 0.26*** | 0.41*** | 0.50*** |  | 0.21*** | 0.24*** | 0.14** |  | -0.03 | -0.13 |
|  | (0.04) |  | (0.04) | (0.07) | (0.04) |  | (0.04) | (0.07) | (0.06) |  | (0.06) | (0.10) |
| unpartnered |  | 1.01*** | 0.97*** | 1.00*** |  | 0.94*** | 0.91*** | 0.92*** |  | 0.53*** | 0.54*** | 0.52*** |
|  |  | (0.03) | (0.03) | (0.03) |  | (0.03) | (0.03) | (0.03) |  | (0.04) | (0.04) | (0.04) |
| childless * unpartnered |  |  |  | -0.26*** |  |  |  | -0.05 |  |  |  | 0.15 |
|  |  |  |  | (0.09) |  |  |  | (0.09) |  |  |  | (0.12) |
| n | 35068 | 35068 | 35068 | 35068 | 35068 | 35068 | 35068 | 35068 | 35068 | 35068 | 35068 | 35068 |

Note: Unweighted estimates. All control variables described in the main documents are included in all models. The four considered models are all fully adjusted for control variables and incrementally add explanatory variables: childlessness (Model 1, M1), unpartnered (Model 2, M2), both (Model 3, M3), both variables and their interaction (Model 4, M4). . Standard errors in parentheses. * p < 0.1; ** p < 0.05; *** p < 0.01.
